# Effectiveness of using group visit model to support diabetes patient self-management in rural communities of Shanghai: a randomized controlled trial

**DOI:** 10.1186/1471-2458-12-1043

**Published:** 2012-12-03

**Authors:** Shengsheng Liu, Anhua Bi, Dongbo Fu, Hua Fu, Wei Luo, Xiaoying Ma, Liyan Zhuang

**Affiliations:** 1 Songjiang District Centre for Disease Prevention and Control, 1050 North Xi Lin Road, Shanghai, 201620, China; 2 Department of Preventive Medicine, School of Public Health, Fudan University, 138 Yi Xue Yuan Road, Shanghai, 20032, China; 3 Fangsong Community Health Centre, Shanghai, China; 4 Zhongshan Community Health Centre, Shanghai, China

**Keywords:** Diabetes, Group visits, Self-management, Community health services, Randomized controlled trials, China

## Abstract

**Background:**

Diabetes has become a major public health problem in China. Support of patient self-management is a key component of effective diabetes care and improved patient outcomes. A series of peer-led community-based disease-specific self-management programs including diabetes have been widely disseminated in urban communities of Shanghai since 1999. However, the strategy of using trained lay leaders to support patient self-management faces challenges in rural communities in Shanghai. This study developed a Chinese diabetes group visit program as an alternative approach to support patient self-management and examined its effectiveness on self-management behaviors, self-efficacy and health status for patients with type 2 diabetes in rural communities of Shanghai.

**Methods:**

208 patients with type 2 diabetes aged 35–80 years were randomly assigned to the intervention group (n=119) of 12 monthly group visit sessions or to a control group (n=89) of usual care. The trial was undertaken in two rural communities in Shanghai, China. Randomization and allocation to study group were carried out by using a random number table. Analysis of covariance was used to compare changes in the 17 self-management behavior, self-efficacy and health status related variables in two groups at 12 months’ follow-up based on 176 patients (n=98; n=78).

**Results:**

Compared with controls, the intervention patients, on average, increased their duration of aerobic exercise by more than 40 minutes per week (*p=0.001*); had significant increase of 0.71 in mean score on self-efficacy to manage diabetes (*p=0.02*); and had significant improvements in measures of illness intrusiveness and systolic blood pressure. The intervention patients attended an average of 10.1 of the 12 program sessions with 75.6% of them attended 10 and more sessions.

**Conclusion:**

The Chinese diabetes group visit model is a feasible, acceptable and effective alternative for supporting diabetes patient self-management in Chinese rural communities. The model requires larger studies to determine its effect on blood glucose control and health care utilization.

**Trial registration:**

ISRCTN87909028

## Background

Diabetes has become a major public health problem in China with 8.2% of rural residents having diabetes [1]. Strategies aimed at improving diabetes care are desperately needed. Support of patient self-management is a key component of effective diabetes care and improved patient outcomes [2,3]. Evidence shows that diabetes self-management education can lead to improvements in outcomes such as glycemic control at least in the short term when delivered in community settings [3-5]. Despite this encouraging evidence, supporting self-management is the least implemented and most challenging area of chronic disease management [6].

In 1999, the authors introduced and tested the Chronic Disease Self-Management Program (CDSMP) - a generic community-based lay-led patient self-management education course developed by Lorig et al. at Stanford University [7]- in Shanghai as a new approach to help people with chronic conditions [8,9]. On the basis of needs assessments, a series of peer-led community-based disease-specific self-management programs including diabetes have been developed, tested and widely disseminated in urban communities of Shanghai in the last ten years [10-14]. More than 1.6 million people attended the chronic disease self-management courses in Shanghai as of the end of 2010 [15]. However, the strategy of using trained lay leaders to support patient self-management faces challenges in rural communities in Shanghai due to inability to recruit enough volunteer lay leaders, community residents' low levels of literacy and their scattered living circumstances [13]. Therefore, exploring alternative strategies to support patient self-management in rural communities is desperately needed.

Self-management can be taught and supported by not only peer leaders, but also health care professionals, office support staff, and other patients [2]. The group visit model, developed in managed care settings to address issues of treatment effectiveness and efficiency, offers promise in improving efficiency and encouraging patient self-management [16,17]. The group visits typically offers patients routine primary care (examinations, diagnoses, and prescriptions) in combination with group support and self-management education [17,18]. Therefore, compared to the peer-led self-management support, providing self-management support in the form of group visits has a stronger linkage to the routine primary care, which may be more acceptable to rural community residents with limited access to health care services.

To date, no other study on group visits has been conducted in the Chinese population. The goal of this study was to explore the group visit model as a new approach to support diabetes patient self-management in rural communities in Shanghai. Our study aimed to develop a Chinese diabetes group visit program and to examine its effectiveness on self-management behaviors, self-efficacy and health status for patients with type 2 diabetes.

## Methods

### Development of the Chinese diabetes group visit program

#### The Chinese diabetes group visit program design and development process

The Chinese diabetes group visit program was developed in three steps. First, selecting an appropriate group visit model. The Cooperative Health Care Clinic Model (the disease specific, patient-focused model) [18] was selected for this study after literature review of evidence and practice implications of current group visit models. Second, qualitative needs assessments of patients with type 2 diabetes and primary care providers on group visit interventions. Four focus group discussions with type 2 diabetes, three focus group discussions with GPs (general practitioners), nurses, and preventive doctors were conducted to collect their suggested content, frequency, group size and format of the group visit program. Finally, a three-day professional workshop was conducted to design the content, format, and protocol for provision of the group visit interventions based on the selected group visit model, the results of the qualitative needs assessments, the Shanghai Community Diabetes Prevention and Control Guidelines [19], the Chinese version of Stanford CDSMP Leader's Manual [20], and the Group Health Cooperative Group Visit Starter Kit [21]. A team of public health experts, GPs, diabetes specialists, and community nurses contributed to planning activities, development of educational materials and the implementation protocol. The final Chinese diabetes group visit program consisted of 12 monthly sessions, groups of 20–25 people met every month over a 12-month period. It used an interactive format to deliver the 12-session self-management education according to the implementation protocol.

#### The structure of the Chinese diabetes group visit program

Each of the 12 sessions of the program was structured into six phases: (1) introduction/feedback; (2) group self-management education; (3) refreshments and group interaction; (4) questions and answers; (5) planning and closing; and (6) one-on-one visits with health care providers. The length of each session was 1.5 hours plus 1 hour post for selected individual visits. The structure of the group visit sessions are summarized in Table 1.

**Table 1:** The template of the Chinese diabetes group visit sessions


15 minutes	**Introduction/Feedback**
	· All team members present introduce themselves and have each participant introduce himself/herself and share two or three problems caused by their diabetes.
	· From the second session, participants will be asked to provide feedback on their action plans made the previous session. If there were problems, the group will be asked to brainstorm possible solutions.
35 minutes	**Topic of group self-management education**
	· Group facilitators will use different adult teaching methods to cover one or two topics each session (Table 2).
15 minutes	**Refreshment and group interaction**
	· Snacks and refreshments provided by volunteers.
	· Ice-breaker games and energizer activities will be used to promote the group interaction.
10 minutes	**Questions and Answers**
	· Participants ask any questions about their diseases, the visit, etc.
	· All team members present will answer the questions related to their area of expertise.
15 minutes	**Planning and Closing**
	· Facilitate each participant to make a weekly action plan for the coming month to achieve their self-management goal.
	· Announce "homework", learning topic, time and date for next session.
60 minutes	**One-on-one visits with health care providers**
	All team members present will meet patients individually as needed for:
	· Measuring and documenting blood pressure, blood glucose and weight
	· Behavioral counseling
	· Refilling prescriptions
	· Ordering referrals, laboratory tests, and treatments as indicated.

#### Topics of the group self-management education

The content and process of the 12 group self-management education sessions were adapted from the Chinese version of Stanford CDSMP Leaders Manual. The content included topics covered in the generic CDSMP course as well as diabetes specific self-management support topics recommended by Shanghai Community Diabetes Prevention and Control Guidelines. Each session had one or two of these topics (see Table 2). Similar to lay-led self-management education programs in Shanghai [8,9,12], these group self-management education sessions focused on helping participants build confidence in their ability to deal with diabetes by incorporating self-efficacy enhancing strategies [22], including action planning and feedback, modeling of behaviors by participants for one another, reinterpretation of symptoms, practicing self-management skills, and group problem-solving. Action plans are designed for one week in lay-led self-management education program [20], but this strategy was modified for each participant to make a weekly action plan for the coming month (four weeks) at each group session in this study. In total, each participant made 12 weekly action plans over the whole 12-month intervention period. In addition, individual learning needs for and barriers to diabetes self-management may not be completely addressed in the group self-management education phase due to time limitations, different learning styles and speeds. Participants could seek further self-management support (more information, knowledge and advice on the issues discussed in the group self-management education phase) during the 60-minute one-on-one visits with health care providers at the end of each group visit session (see Table 1). In this study, about one-fourth of participants eventually received this additional self-management support individually in each group visit session.

**Table 2:** Topics in each of the 12 group self-management education sessions

**Session**	**Topics**
Session 1	Overview of self-management and diabetes
	Setting goals and making an action plan
Session 2	Relaxation/cognitive symptom management
Session 3	Increasing aerobic exercise
Session 4	Healthy eating
	Meal planning
Session 5	Managing medications
	Insulin injection
Session 6	Fatigue management
	Dealing with anger/fear/frustration
Session 7	Routine medical checkups
	Understanding the results of blood tests
Session 8	Preventing and treating acute hypoglycemia
Session 9	Hypertension management
Session 10	Diabetic foot care
Session 11	Communicating and working as a partner with the healthcare team
	Seeking support from family and friends
Session 12	Planning for the future

### Implementation of the Chinese diabetes group visit program

The Chinese diabetes group visit program was implemented in two rural communities in Songjiang District, Shanghai from June 2007 to May 2008 by three existing general practice teams consisted of one GP, one preventive doctor and one nurse practitioner. The team members had identical roles as group facilitators. They all attended a one-day training workshop with the focus on adult teaching methods used in the CDSMP course. They alternated leading the 12 group self-management education sessions based on their areas of expertise. Each group also had one group leader who himself or herself had diabetes, lived in the same community of other group members, and volunteered to lead the group. The group leader played several important roles in implementing the program, including reminding the group members about upcoming group visit, arranging volunteers to bring snacks and refreshments, and following up with group members on their action plans in person or by telephone within one week.

Based on the successful experiences of the community-based lay-led self-management programs in Shanghai [8,9], the "commonly participatory model" was used to implement this program, in which the community government, community health centre, Songjiang District Centre for Disease Prevention and Control (CDC) and researchers from School of Public Health, Fudan University worked together to conduct and evaluate the program.

### Patients

The study participants were 937 patients with type 2 diabetes who lived in two rural communities in Songjiang District, Shanghai and were included in Songjiang District CDC's diabetes patient registration database. The inclusion criteria were men and women aged 35–80 with type 2 diabetes confirmed by medical records, and community dwelling. Patients aged less than 35 years or more than 80 years, patients with mental illness or stroke patients with severe physical disability that would prevent attendance or participation in the groups were not contacted for the study. The Ethics Committee of the School of Public Health, Fudan University approved this study, and written consent form was obtained for each participating patient.

### Recruitment and randomization

891 (95.1%) of 937 patients with type 2 diabetes who met the inclusion criteria were identified using the computer system. They were contacted by a recruitment letter describing the diabetes group visit intervention as a "new community diabetes care service provided by the community health centre", where the participant would "learn how to self-manage their diabetes in a group led by a general practice team every month in the community". In addition, the diabetes group visit program was also announced as a new diabetes care delivery model to increase public awareness in the two rural communities through advertisements in the mass media, posters at community health centres. 208 patients (23% of 891 eligible patients) eventually participated in this study.

**Figure 1:**
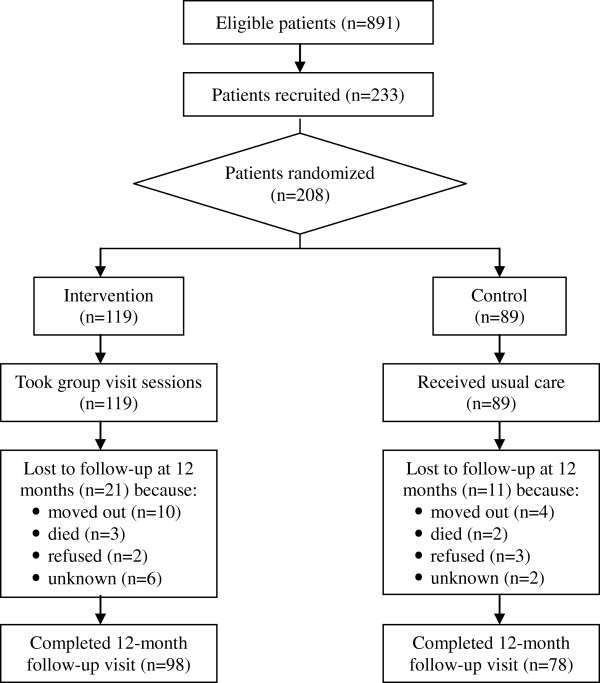
Flow diagram of participants.

Randomization was conducted at each of the two rural communities separately after each participant completed a baseline questionnaire and signed an informed consents form. Participants in the same community were randomized into "intervention group" and "control group", according to a random-number table with a randomization ratio designed to yield no fewer than 20 and no more than 25 participants in a group. Investigators and assessors (those collecting and analyzing data) were blinded to group assignments. Participants were aware of their treatment assignments. The members of the general practice care team knew which participants were in intervention group, but they did not know which patients in the community served as control subjects for this study. As a result, 119 patients were placed in intervention group and 89 patients in control group. The intervention group received the 12-session diabetes group visit interventions from June 2007 to May 2008. Control group members received usual care by a single general practitioner on a one-to-one basis. Usual care included one or more of the following depending on the needs of the patient: measuring blood pressure, blood glucose and weight; counseling on diet management, exercise and medication use; refilling prescriptions; and ordering referrals, laboratory tests, and treatment of diabetic complications. Figure 1 shows the results of the patient recruitment and the trial profile.

### Data collection and statistical analysis

The main outcome variables included: (1) self-management behavior scores (diet, aerobic exercise, practice of cognitive symptom management, communication with doctor, and examining feet); (2) self-efficacy score (self-efficacy is defined here as perceived level of confidence in one’s ability to manage diabetes in general); and (3) health status (self-rated health, energy, health distress, level of fatigue, illness intrusiveness, depression, body mass index, systolic and diastolic blood pressure levels). With the exception of the questions about diet, examining feet, body mass index (BMI), and blood pressure levels, which were developed for this study, all measures had been previously validated [12]. The self-efficacy score was measured by using the Chinese version of the 8-item Diabetes Self-efficacy Scale developed by Stanford Patient Education Research Center [23]. The questionnaire was completed by participants at baseline and 12 months later. 208 participants completed a baseline questionnaire, and 176 of them (98 in intervention group, 78 in control group) also completed a 12-month follow-up questionnaire. Data collection was completed by university students who did not know the patients or their intervention status.

Descriptive statistics were used to document the demographics and comorbidity of the study participants. Chi square test and analysis of variance were used to detect the difference of demographic data between groups. The Mann–Whitney U-test was used to compare the baseline status of the intervention and control groups. Analysis of covariance was used to compare changes in the 17 outcome variables at 12 months between the intervention and control groups. The analysis controlled for age, sex, education, marital status, and the baseline value of the study variables that differed between the groups at baseline: hypertension, baseline level of fatigue and illness intrusiveness.

## Results

### Baseline characteristics

208 out of 891 eligible patients eventually participated in this study. Compared with all 891 eligible patients contacted, those who were recruited had a similar sex distribution and mean level of education, but a significantly higher mean age and prevalence of hypertension. Table 3 shows demographics and comorbidity of 208 patients who participated in the study. Only prevalence of hypertension was significantly different between those in the intervention and control groups (*p=0.02*). Table 4 gives baseline data for the intervention and control groups. A comparison of baseline data showed that patients in intervention group had significantly better (lower scores for) fatigue and illness intrusiveness than patients in control group (*p <0.05*).

**Table 3:** Patient characteristics. Values are numbers (percentages) unless otherwise specified

**Characteristic**	**Intervention (n=119)**	**Control (n=89)**
**Mean ± SD**^**a**^**age (years)**	61.99 ± 9.80	62.49 ± 9.97
**Sex**		
Female	74 (62.2)	55 (61.8)
Male	45 (37.8)	34 (38.2)
**Ethnicity**		
Han	113 (95.0)	87 (97.8)
Zhuang	5 (4.2)	1 (1.1)
Other	1 (0.8)	1 (1.1)
**Mean ± SD education (years)**	6.22 ± 4.43	6.08 ± 4.77
**Marital status**		
Married	103 (86.6)	78 (87.7)
Separated	1 (0.8)	1 (1.1)
Widowed	14 (11.8)	10 (11.2)
Single	1 (0.8)	0 (0.0)
**Comorbidity**		
Hypertension	75 (63.0) ^b^	42 (47.2)
Heart disease	14 (11.8)	7 (7.9)
Stroke	2 (1.7)	1 (1.1)
Nephropathy	2 (1.7)	1 (1.1)
Retinopathy	21 (17.6)	12 (13.5)
Diabetic foot	1 (0.8)	0 (0.0)
Neuropathy	8 (6.7)	2 (2.2)

^a^ SD = standard deviation. ^b^*χ*^*2*^=51.19, *p*=0.02.

**Table 4:** Baseline for intervention and control patients: self-management behavior, self-efficacy and health status

**Variable**	**Mean ± SD**		***P*-value**^**a**^
	**Intervention (n = 119)**	**Control (n = 89)**	
**Self-management behavior**			
Aerobic exercise (minutes/week)	153.78 ± 154.93	129.10 ± 125.98	0.48
Eating fatty foods (grams per day)	179.54±130.50	177.18 ±133.05	0.12
Eating fruits (pieces per day)	0.83 ± 0.61	0.78 ± 0.73	0.17
Eating vegetables (grams per day)	221.40 ± 117.82	210.67 ± 138.57	0.38
Cognitive symptom management^b^	0.78 ± 0.81	0.95 ± 0.88	0.14
Communication with medical doctor^b^	1.90 ± 1.24	1.69 ± 0.97	0.40
Examining feet (times per week)	1.05 ± 1.93	1.09 ± 2.22	0.21
**Self-efficacy to manage diabetes**^c^	8.03 ± 1.95	8.10 ± 1.88	0.89
**Health status**			
Self-rated health^d^	3.69 ± 0.71	3.78 ± 0.54	0.54
Energy^b^	2.61 ± 0.94	2.62 ± 0.81	0.73
Health distress^e^	0.96 ± 1.03	0.90 ± 0.93	0.95
Fatigue^f^	2.79 ± 2.16	3.43 ± 1.86	0.01
Illness intrusiveness^g^	25.34 ± 12.76	30.60 ± 16.37	0.03
Depression^h^	6.87 ± 4.52	7.60 ± 4.64	0.40
BMI	23.96 ± 3.28	23.72 ± 3.01	0.59
Systolic blood pressure (mmHg)	129.90 ± 11.39	128.83 ± 11.74	0.51
Diastolic blood pressure (mmHg)	78.49 ± 7.03	77.97 ± 6.31	0.96

^a^ Mann–Whitney U-test was used to compare baseline variables between intervention and control groups (two-tailed *P*-values).

^b^ 0–5 increase = improvement.

^c^ 1–10 increase = improvement.

^d^ 1–5 decrease = improvement.

^e^ 0–5 decrease = improvement.

^f^ 0–10 decrease = improvement.

^g^ 13–91 decrease = improvement.

^h^ 0–30 decrease = improvement.

### Effectiveness of the Chinese diabetes group visit interventions

Table 5 shows the mean changes in self-management behavior, self-efficacy, and health status for 176 patients who completed both baseline and 12-month follow up questionnaires. Compared with controls, patients in intervention groups had significant improvement in one self-management behavior, self-efficacy to manage diabetes, and two of the nine health status indicators after controlling for co-variables. Patients in intervention group, on average, increased their duration of aerobic exercise by more than 40 minutes per week (*p=0.001*). The intervention group had significant increase of 0.71 in mean score on self-efficacy to manage diabetes (*p=0.02*). Patients in intervention group also had significant improvements in measures of illness intrusiveness and systolic blood pressure. The intervention group had, on average, 3.72 mmHg fewer increase in systolic blood pressure (*p=0.04*).

**Table 5:** 12-month changes for intervention and control patients: self-management behavior, self-efficacy and health status

**Variable**	**Mean ± SD**		***P*-value**^**a**^
	**Intervention (n = 98)**	**Control (n = 78)**	
**Self-management behavior**			
Aerobic exercise (minutes/week)	23.11 ± 176.71	−18.27 ± 156.22	0.001
Eating fatty foods (grams per day)	−0.06 ±15.76	0.92 ±6.60	0.14
Eating fruits (pieces per day)	0.12 ± 0.61	0.16 ± 0.91	0.51
Eating vegetables (grams per day)	22.94 ± 154.43	−10.26 ± 165.75	0.60
Cognitive symptom management^b^	0.37 ± 1.06	0.03 ± 1.16	0.10
Communication with medical doctor^b^	0.41 ± 1.54	0.22 ± 1.09	0.97
Examining feet (times per week)	0.46 ± 2.08	0.45 ± 2.86	0.76
**Self-efficacy to manage diabetes**^c^	0.18 ± 2.24	−0.53 ± 1.96	0.02
**Health status**			
Self-rated health^d^	−0.04 ± 0.82	0.12 ± 0.70	0.10
Energy^b^	−0.16 ± 1.18	−0.07 ± 1.01	0.20
Health distress^e^	0.04 ± 1.26	0.14 ± 1.07	0.78
Fatigue^f^	0.45 ± 2.49	0.35 ± 2.30	0.39
Illness intrusiveness^g^	2.50 ± 15.68	6.81 ± 18.20	0.001
Depression^h^	4.49 ± 4.99	3.92 ± 5.01	0.43
BMI	0.06 ± 1.14	0.28 ± 1.26	0.22
Systolic blood pressure (mmHg)	1.48 ± 12.03	5.20 ± 12.34	0.04
Diastolic blood pressure (mmHg)	1.45 ± 8.86	2.65 ± 7.72	0.54

^a^ Analysis of covariance on 12-month post-test scores controlling for the baseline value of study variables, age, sex, education, marital status, hypertension, and baseline number of fatigue and illness intrusiveness (two-tailed *P*-values).

^b^ 0–5 increase = improvement.

^c^ 1–10 increase = improvement.

^d^ 1–5 decrease = improvement.

^e^ 0–5 decrease = improvement.

^f^ 0–10 decrease = improvement.

^g^ 13–91 decrease = improvement.

^h^ 0–30 decrease = improvement.

## Discussion

Our study is the first one to test the effectiveness of self-management support through group visit model for Chinese people with type 2 diabetes. The content of the group self-management education in the Chinese diabetes group visit program was adapted from the Chinese version of Stanford CDSMP course, which has proved to be culturally acceptable to Chinese people [8-14]. Support from group leaders and other group members on action plan implementation was identified by participants as one of the most liked aspects of the CDSMP course in Shanghai [9]. Building on this experience, recruiting a volunteer group leader to support group members in adhering their action plans was included as a unique component of the Chinese diabetes group visit program. The group leader played an important role in following up with group members on their action plans as they lived in the same community and had peer contact on a regular basis. Program implementation was integrated into the routine of community health services and general practice team.

Similar to the lay-led generic CDSMP and Diabetes Self-Management Program in urban communities of Shanghai [8,12], the results of this study showed that the group visit model was effective in increasing self-management behavior (aerobic exercise), self-efficacy, maintaining and improving health status (illness intrusiveness and systolic blood pressure levels) in patients with type 2 diabetes (Table 5). Our findings that the average minutes of aerobic exercise per week and the mean systolic blood pressure levels of the intervention patients were significantly improved at 12 months, are in accordance with findings from other group visit studies for adults with type 2 diabetes [24,25].

It is important to note that group visit model appears to be a feasible and acceptable method for supporting diabetes patient self-management in Chinese rural communities according to the results of the patient recruitment and attendance status in this study. The recruitment rate of this study was 23% (208 out of 891 eligible patients), which is similar to the lay-led Diabetes Self-Management Program in urban communities of Shanghai [12]. The recruitment rate of this study is higher than the response rates of those group visit studies that used the same recruitment technique [17], but lower than the 50% recruitment rate of the study that used an active recruitment strategy such as encouragement from the physician [26]. The intervention patients attended an average of 10.1 of the 12 program sessions with 75.6% of them attended 10 and more sessions, suggesting that they were highly motivated to participate. Compared to an average of 6.0 of the seven sessions in our lay-led generic CDSMP in urban communities in Shanghai [8,9], this program had similar success.

Limitations of this study need to be considered in interpreting the results. Firstly, the patients who actually participated in this study were significantly older, with a higher prevalence of hypertension than the patients who declined to participate. Therefore, the outcomes may not necessarily apply to younger patients with type 2 diabetes who are not accompanying by hypertension in Chinese rural communities. This suggests that research on group visits in Shanghai in the future may need to explore effective strategies to motivate younger patients and those not accompanying by hypertension to attend. Secondly, this study had a relatively small sample size, which prevented quantitatively evaluating the effect of the group visit model on blood glucose levels, HbA1c and health care utilization. Thirdly, nearly 15% of participants did not complete the study, which may cause bias results.

## Conclusion

This study shows that the Chinese diabetes group visit model is a feasible, acceptable and effective alternative for providing self-management support to patients with type 2 diabetes in Chinese rural communities. The model has the potential of reaching a relatively large number of patients at a low cost through a mass mailing invitation and community public service announcements in Chinese rural communities. The model was well-accepted by study participants. The Chinese diabetes group visit model has benefits similar to those reported in the studies of Shanghai peer-led patient self-management education programs and other diabetes group visit studies in developed countries in improving participants' self-management knowledge and behavior, self-efficacy and health status. The effect of the Chinese diabetes group visit model on blood glucose levels, HbA1c, health care utilization and practice efficiency, needs further study with a larger sample size.

## Abbreviations

CDSMP: Chronic Disease Self-Management Program; GPs: General practitioners; CDC: Centre for Disease Prevention and Control; BMI: Body mass index.

## Competing interests

The authors declare that they have no competing interests.

## Author’s contributions

SL conceived of the study and drafted the manuscript. AB participated in the study design and coordination. DF helped to conceptualize the study, conducted the statistical analysis, and helped to draft the manuscript. HF participated in the study design and contributed to the interpretation of results. WL, XM and LZH oversaw the data collection and assisted in the implementation of the study. All authors contributed to critical revision of and approved the final manuscript.

## Pre-publication history

The pre-publication history for this paper can be accessed here:

http://www.biomedcentral.com/1471-2458/12/1043/prepub
